# Efficacy and safety of calcitonin-gene-related peptide binding monoclonal antibodies for the preventive treatment of episodic migraine – an updated systematic review and meta-analysis

**DOI:** 10.1186/s12883-020-01633-3

**Published:** 2020-02-15

**Authors:** Hong Deng, Gai-gai Li, Hao Nie, Yang-yang Feng, Guang-yu Guo, Wen-liang Guo, Zhou-ping Tang

**Affiliations:** Department of Neurology, Tongji Hospital, Tongji Medical college, Huazhong University of Science and Technology, Wuhan, 430030 China

**Keywords:** Calcitonin gene-related peptide monoclonal antibody, Episodic migraine, Efficacy, Safety, Meta-analysis

## Abstract

**Background:**

Migraine is one of the most common neurological disorders that leads to disabilities. However, the conventional drug therapy for migraine might be unsatisfactory at times. Therefore, this meta-analysis aimed to evaluate the efficacy and safety of calcitonin-gene-related peptide binding monoclonal antibody (CGRP mAb) for the preventive treatment of episodic migraine, and provide high-quality clinical evidence for migraine therapy.

**Methods:**

A systematic electronic database search was conducted to identify the potentially relevant studies. Two independent authors performed data extraction and quality appraisal. Mean difference (MD) and risk ratio (RR) were pooled for continuous and dichotomous data, respectively. The significance levels, weighted effect sizes and homogeneity of variance were calculated.

**Results:**

Eleven high-quality randomized control trials that collectively included 4402 patients were included in this meta-analysis. Compared to placebo group, CGRP mAb therapy resulted in a reduction of monthly migraine days [weighted mean difference (WMD) = − 1.44, 95% CI = (− 1.68,− 1.19)] and acute migraine-specific medication days [WMD = − 1.28, 95% CI = (− 1.66,− 0.90)], with an improvement in 50% responder rate [RR = 1.51, 95% CI = (1.37,1.66)]. In addition, the adverse events (AEs) and treatment withdrawal rates due to AEs were not significantly different between CGRP mAb and placebo groups. Similar efficacy and safety results were obtained for erenumab, fremanezumab, and galcanezumab in subgroup analysis.

**Conclusions:**

The current body of evidence reveals that CGRP mAb is an effective and safe preventive treatment for episodic migraine.

## Background

Migraine is one of the most common neurological diseases characterized by unilateral localization, pulsating quality, moderate to severe pain intensity and avoidance of movement [1, 2]. According to the 2013 Global Burden of Disease Study, over half of all years lost to disability resulting from neurological disorders are attributed to migraine [3–5]. Episodic migraine is the most common form of migraine, defined as occurring on fewer than 15 days per month in accordance with the third version of the International Classification of Headache Disorders (ICHD-3) edited by the International Headache Society (IHS) [6, 7]. It can be further subdivided into high-frequency episodic migraine (HFEM) and low-frequency episodic migraine (LFEM) based on frequency. Previous studies usually used frequencies from 8 to 14 and 10 to 14 migraine headache days (MHDs) per month to define HFEM [8]. As for when to start preventive treatment, there is no certain evidence now, only based on rules of thumb or expert opinions [9–11]. It may depend on a number of factors, including attack frequency and severity, responsiveness to medications for acute migraine, and coexisting conditions [9]. It’s generally believed that preventive therapy should be initiated if migraine occurs at least once per week or on 4 or more days per month [9]. However, due to the lack of efficacy and intolerable side effects of available conventional preventive therapies, the management of patients with migraine might be unsatisfactory sometimes. Thus, novel effective drugs with good tolerability, few side effects and high retention rates are needed for episodic migraineurs.

Calcitonin gene-related peptide (CGRP) has been found to play an important role in the pathophysiology of migraine via nociceptive mechanisms in the trigeminovascular system [12]. At present, there are four monoclonal antibodies (mAbs) targeting the CGRP, namely, eptinezumab (ALD403), fremanezumab (TEV-48125; previously known as LBR-101 or RN-307), galcanezumab (LY2951742) and erenumab (AMG334). The former three are humanized mAbs that potently and selectively bind to CGRP, while the latter one is the only monoclonal antibody that targets CGRP receptor instead of CGRP ligand. All of them have been studied in clinical trials for the preventive treatment of episodic migraine.

Although a previous meta-analysis has assessed the efficacy and safety of CGRP mAbs for episodic migraine [13], several new high-quality randomized control trials (RCTs) are not included in the published meta-analysis [14–18]. Therefore, we conducted an updated meta-analysis to comprehensively investigated the efficacy and safety of CGRP mAbs for the preventive treatment of episodic migraine.

## Methods

### Literature search

This meta-analysis was conducted according to the recommendations of the Preferred Reporting Items for Systematic Reviews and Meta-Analyses (PRISMA) statement. We systematically searched MEDLINE, EMBASE, the Cochrane Controlled Trials Register (CENTRAL), and Web of Science (from inception to 9th, March,2019). The search keywords included (“eptinezumab” OR “ALD403” OR “fremanezumab”OR “TEV-48125” OR “galcanezumab” OR “LY2951742” OR “erenumab” OR “AMG334”) AND “episodic migraine”. There were no area limitation or language restriction. To identify other potentially relevant studies, the reference lists of the retrieved articles were searched manually.

### Study selection

Studies were included in this meta-analysis if they met the following criteria. (i) Randomized, double-blinded, placebo-controlled, parallel-group studies with experimental and control groups receiving CGRP mAbs and matched placebo, respectively. (ii) Adults aged ≥18 years, regardless of gender or ethnicity. (iii) Subjects diagnosed with episodic migraine according to the International Classification of Headache Disorders III (ICHD-III) for at least 1 year prior to enrollment [19]. (iv) Studies reported at least one of the following outcomes: the decreased number of monthly migraine days, ≥ 50% reduction from baseline in the mean number of migraine days per month, monthly acute migraine-specific medication prescribed from baseline to endpoint, and adverse events (AEs).

Exclusion criteria were: (i) non-human studies; (ii) case series or case reports; (iii) review articles, meta-analysis or letters to the editor; and (iv) multiple reports from the same cohort.

One author (HD) performed initial eligibility screening by assessing the titles and abstracts of all retrieved articles. Following initial screening, 2 authors (HD and G-GL) independently reviewed the full-text copies of potentially eligible articles. Disagreements were resolved through discussion.

### Outcome measurement

The primary efficacy outcome measures were the changes in the number of monthly migraine days from baseline to endpoint and monthly acute migraine-specific medication days. We extracted the data at weeks 9–12 in most time. If the data was not available, those at week 24 were used instead [17, 18]. The achievement of at least a 50% reduction from baseline in the mean number of migraine days per month was assessed as the secondary efficacy outcome. The primary safety outcome was the proportion of participants who suffered adverse events (AEs). The proportions of patients who withdrew from treatment due to AEs and experienced any serious AEs (SAEs) were also assessed. If more than two dosages were used in a single RCT, the outcome values of the most common dosage group were pooled for each type of CGRP mAbs. However, if only one dosage was reported in a single RCT, the outcome values of that dosage were analyzed.

### Risk of Bias assessment

The Cochrane Collaboration’s tool was used to assess the risk of bias. Two authors (DH and G-GL) independently judged whether the risk of bias for each criterion was considered low, high or unclear. Disagreements were resolved by discussion.

### Statistical analysis

The heterogeneity between trials was examined using the I^2^ statistic. For continuous and dichotomous outcome data, the mean difference (MD) and risk ratio (RR) with 95% confidence intervals (CIs) were respectively calculated. In the case of only one available study, we calculated only the MD in migraine frequency or RR for response to treatment. All analyses were carried out using the Review Manager (RevMan 5.3; The Nordic Cochrane Centre, The Cochrane Collaboration, Copenhagen, Denmark). Publication bias was assessed through visual inspection of the funnel plots. Trial sequential analysis (TSA, version 0.9.5.10 Beta, http:// www.ctu.dk/tsa/downloads.aspx) was managed to evaluate the cumulative evidence according to the information size achieved to date.

## Results

### Eligible studies

**Fig. 1:**
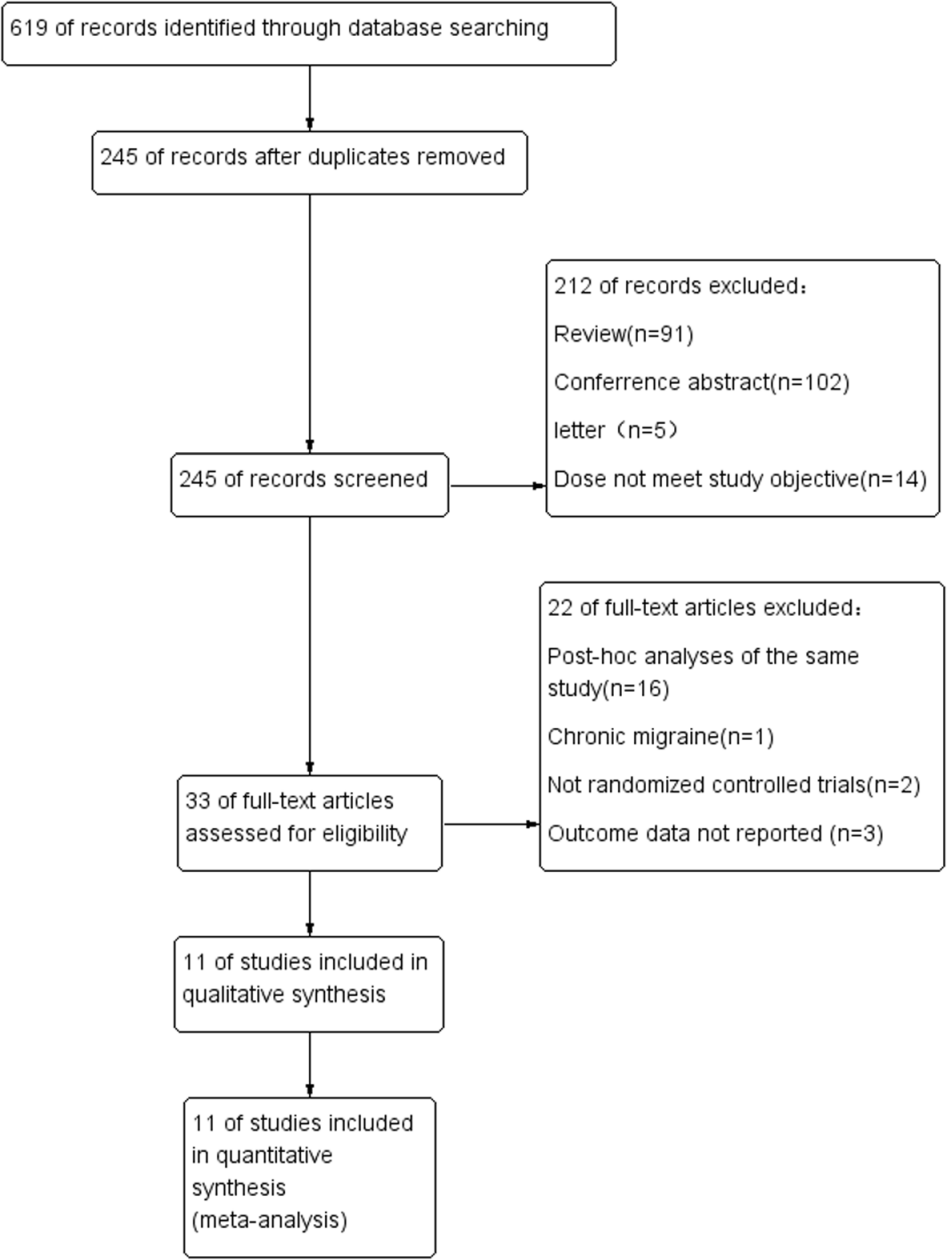
Flow diagram of study selection process

Six hundred and nineteen records were identified through database and trial registry searching. After excluding the conference abstracts, reviews, letters and irrelevant studies by screening the titles or abstracts, a total of 33 full texts were retrieved for more detailed inspection. Sixteen of them were repeated publication or post-hoc analysis of the same study and two of them were not RCTs. In addition, 4 articles were excluded for the reasons of chronic migraine [20], healthy subjects [21, 22] or without placebo group [23]. Finally, a total of 11 studies met the inclusion and exclusion criteria [14–18, 24–29], and at least 1 outcome could be included in this meta-analysis (Fig. 1).

### Characteristics of the included studies

Eleven studies with data from 4402 unique participants were included. All the included studies were multi-center, randomized, double-blind, placebo-controlled trials involving 5 phase II [25–29] and 6 phase III trials [14–18, 24]. A phase III RCT, namely, PROMISE-1 (NCT02559895), was excluded due to the unpublished original data [30]. Data with the usage of erenumab (70 mg per month), eptinezumab (1000 mg per month), fremanezumab (225 mg per month) and galcanezumab (120 mg per month) were selected for pooled analysis. One RCT contained only the dosage group of 140 mg erenumab was included [14]. For galcanezumab, we included a study with the dosage of 150 mg per month, which was relatively close to 120 mg per month [29]. The age of episodic migraine sufferers ranged between 18 and 70 years. Most of the double-blind, placebo controlled trials lasted for 12 weeks, except for three studies with 24 weeks [17, 18, 24]. Detailed characteristics of the included study are shown in Table 1. According to the Cochrane Handbook of Systematic Review, the risks of bias were assessed (Table 2).

**Table 1:** Characteristics of the included studies

Study (reference no.)	Year	Study design (NCT No.)	Interventions	Sex (male/female),Age (mean ± SD)	Baseline Migraine-days per month (mean ± SD)	Follow-up
Uwe Reuter [14]	2018	RCT phase3b, NCT03096834	erenumab 140 mg Placebo	24/97,44.6 ± 10.5 22/103,44.2 ± 10.6	9.2 ± 2.6 9.3 ± 2.7	12w
David W Dodick [15]	2017	RCT phase 3, NCT02483585	erenumab 70 mg Placebo	41/245,42 ± 11 44/247,42 ± 12	8.1 ± 2.7 8.4 ± 2.6	12w
Peter J. Goadsby [24]	2017	RCT phase 3, NCT02456740	erenumab 70 mg Placebo	49/268,41.1 ± 11.3 45/274,41.3 ± 11.2	8.3 ± 2.5 8.2 ± 2.5	24w
Hong Sun [25]	2016	RCT phase 2, NCT01952574	erenumab 70 mg Placebo	25/82, 42.6 ± 9.9 28/132,41.4 ± 10.0	8.6 ± 2.5 8.8 ± 2.7	12w
David W Dodick [26]	2014	RCT phase 2, NCT01772524	Eptinezumab 1000 mg Placebo	14/67,38.6 ± 10.8 16/66,39.0 ± 9.6	8.4 ± 2.1 8.8 ± 2.7	12w
David W. Dodick [16]	2018	RCT phase 3, NCT02629861	Fremanezumab 225 mg Placebo	46/244,42.9 ± 12.7 47/247, 41.3 ± 12.0	8.9 ± 2.6 9.1 ± 2.7	12w
Marcelo E Bigal [27]	2015	RCT phase 2b, NCT02025556	Fremanezumab 225 mg Placebo	9/87,40.8 ± 12.4 12/92,42.0 ± 11.6	11.5 ± 1.9 11.5 ± 2.24	12w
Vladimir Skljarevski^**#**^ [28]	2018	RCT phase 2b, NCT02163993	Galcanezumab 120 mg Placebo	42/231,40.6 ± 11.9 28/109,39.5 ± 12.1	6.7 ± 2.6 6.6 ± 2.7	12w
Vladimir Skljarevski [18]	2017	RCT Phase 3, NCT02614196	galcanezumab 120 mg Placebo	34/197,40.9 ± 11.2 68/393,42.3 ± 11.3	9.07 ± 2.9 9.2 ± 3.0	24w
Virginia L. Stauffer [17]	2018	RCT phase 3, NCT02614183	galcanezumab 120 mg Placebo	32/181,40.9 ± 11.9 71/362,41.3 ± 11.4	9.2 ± 3.1 9.1 ± 3.0	24w
David W Dodick [29]	2014	RCT phase 2, NCT01625988	galcanezumab 150 mg Placebo	19/88,40.9 ± 11.4 14/96,41.9 ± 11.7	6.7 ± 2.4 7.0 ± 2.5	12w

*RCT* Randomized controlled trial, *SD* Standard deviation. #The specific information can only be achieved in the total CGRP monoclonal antibodies treatment group

**Table 2:** Assessment on the methodological strategies of the included studies

Trial ID	Random sequence generation	Allocation concealment	Blinding	Incomplete outcome data	Selective outcome reporting	Other sources of bias
Uwe Reuter 2018	Low risk	Low risk	Low risk	Low risk	Low risk	Unclear risk
David W Dodick 2017	Low risk	Low risk	Low risk	Low risk	Low risk	Unclear risk
Peter J. Goadsby 2017	Low risk	Low risk	Low risk	Low risk	Low risk	Unclear risk
Hong Sun 2016	Low risk	Low risk	Low risk	Low risk	Low risk	Unclear risk
David W Dodick 2014	Low risk	Low risk	Low risk	Low risk	Low risk	Unclear risk
David W. Dodick 2018	Low risk	Low risk	Low risk	Low risk	Low risk	Unclear risk
Marcelo E Bigal 2015	Low risk	Low risk	Low risk	Low risk	Low risk	Unclear risk
Vladimir Skljarevski 2018	Low risk	Low risk	Low risk	Low risk	Low risk	Unclear risk
Vladimir Skljarevski 2017	Low risk	Low risk	Low risk	Low risk	Low risk	Unclear risk
Virginia L. Stauffer 2018	Low risk	Low risk	Low risk	Low risk	Low risk	Unclear risk
David W Dodick 2014	Low risk	Low risk	Low risk	Low risk	Low risk	Unclear risk

### Monthly migraine days

**Fig. 2:**
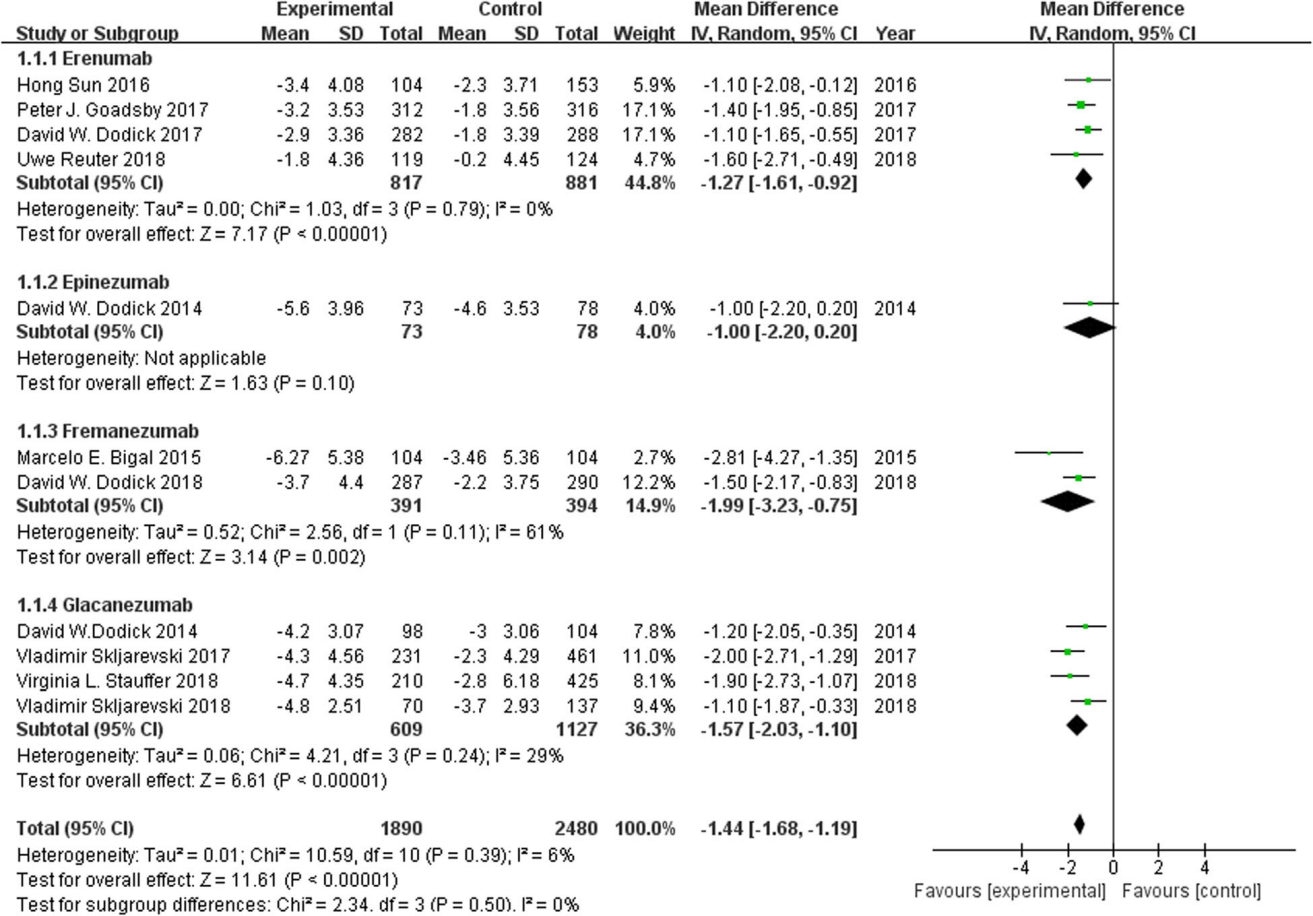
Forest plot of CGRP mAb vs. placebo for the changes in baseline monthly migraine days. The estimated pooled WMD was − 1.44 (95% CI, − 1.68 to − 1.19) with high statistical significance (*P* < 0.00001). There was low heterogeneity among the studies (I^2^ = 6%). *SD standard deviation, CI confidence interval, WMD weighted mean difference*

All the 11 trials reported the changes in monthly migraine days from baseline to endpoint. It was found that erenumab, fremanezumab and galcanezumab exhibited significant differences in this clinical index as compared to placebo group (MD -1.27, 95% CI − 1.61 to − 0.92; MD -1.99, 95% CI − 3.23 to − 0.75; and MD -1.57, 95% CI − 2.03 to − 1.10, respectively). After pooling, the change in monthly migraine days from baseline to endpoint was significantly greater for CGRP mAbs compared to placebo [weighted mean difference (WMD) = − 1.44, 95% CI = (− 1.68, − 1.19), I^2^ = 6%, *p* < 0.00001]. The results are demonstrated in Fig. 2.

### Monthly acute migraine-specific medication days

**Fig. 3:**
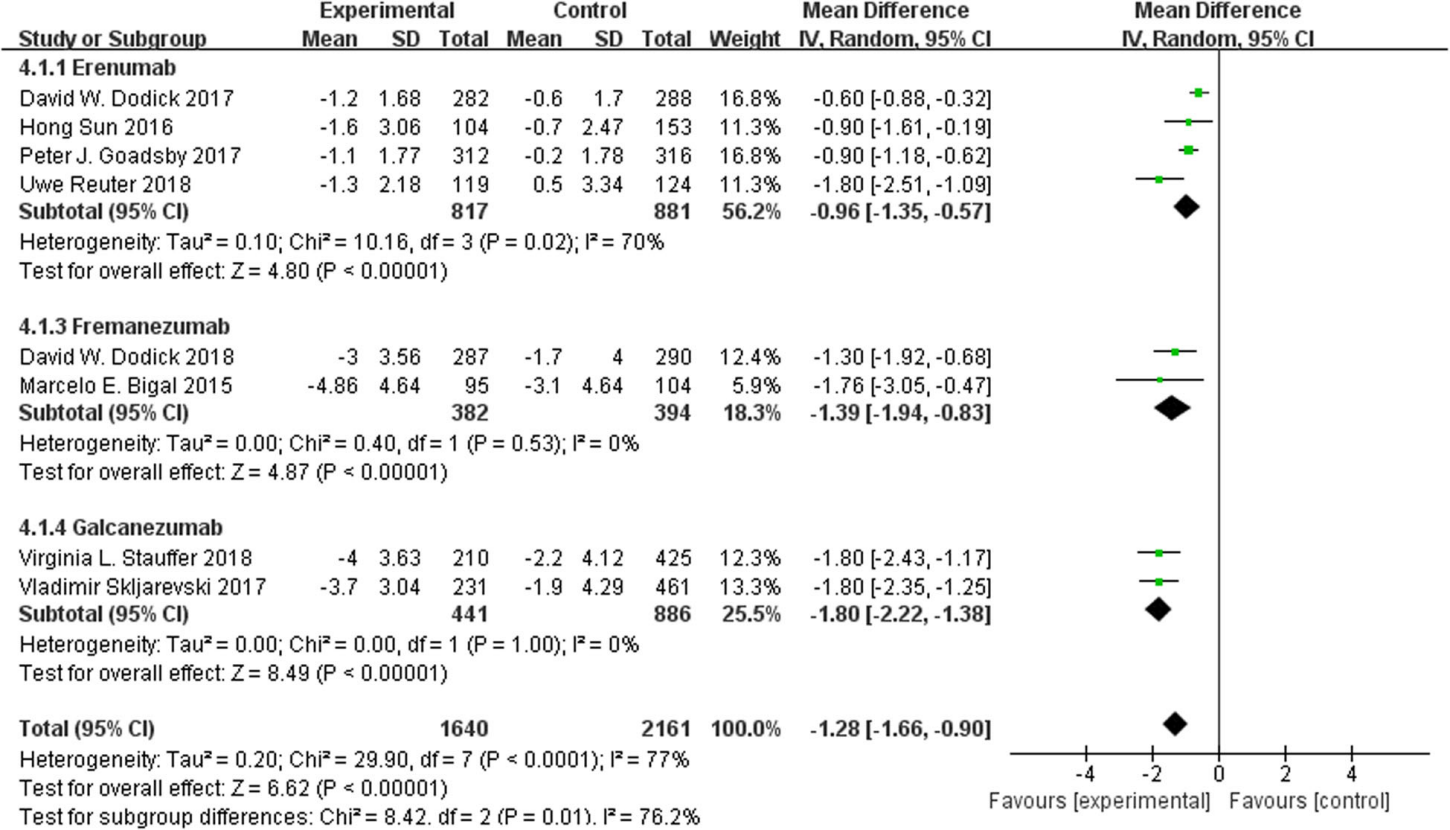
Forest plot of CGRP mAb vs. placebo for the changes in baseline monthly acute migraine-specifc medication days. The estimated pooled WMD was − 1.28 (95% CI, − 1.66 to − 0.90) with high statistical significance (*P* < 0.00001). There was high heterogeneity among the studies (I^2^ = 77%). *SD* standard deviation, *CI* confidence interval, *WMD* weighted mean difference

Eight trials reported the changes in monthly acute migraine-specific medication days from baseline to endpoint. It was found that erenumab, fremanezumab and galcanezumab exhibited significant differences in this clinical index as compared to placebo group (MD -0.96, 95% CI − 1.35 to − 0.57; MD -1.39, 95% CI − 1.94 to − 0.83; and MD -1.80, 95% CI − 2.22 to − 1.38, respectively). After pooling, the change in monthly acute migraine-specific medication days from baseline to endpoint was significantly greater for CGRP mAbs compared to placebo (WMD = − 1.28, 95% CI = [− 1.66, − 0.90], I^2^ = 77%, *p* < 0.00001). The results are presented in Fig. 3.

### ≥ 50% reduction from baseline in monthly migraine days

**Fig. 4:**
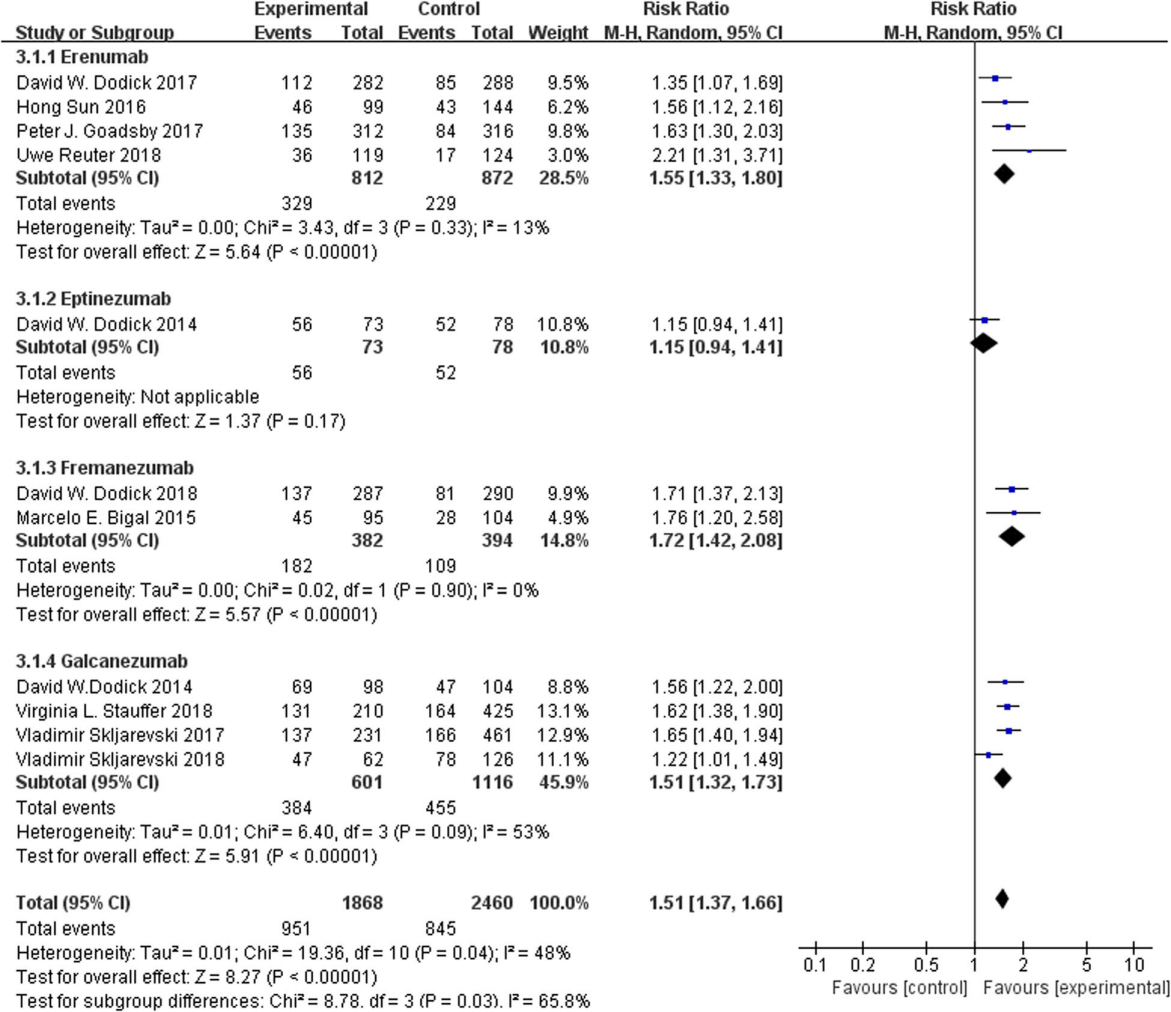
Forest plot of CGRP mAb vs. placebo for the reduction of 50% responder rates. The estimated pooled RR was 1.51 (95% CI, 1.37 to 1.66) with high statistical significance (*P* < 0.00001). There was moderate heterogeneity among the studies (I^2^ = 48%). *CI* confidence interval, *RR* risk ratio

All the 11 trials reported the 50% responder rate. It was observed that erenumab, fremanezumab and galcanezumab exhibited significant differences in this clinical index as compared to placebo group (RR 1.55, 95% CI 1.33 to 1.80; RR 1.72, 95% CI 1.42 to 2.08; and RR 1.51, 95% CI 1.32 to 1.73, respectively). After pooling, the change in ≥50% reduction in migraine days per month from baseline to endpoint was remarkably greater for CGRP mAbs compared to placebo (RR = 1.51, 95% CI = [1.37, 1.66], I^2^ = 48%, *p* < 0.00001). The results are shown in Fig. 4.

### Adverse events

**Fig. 5:**
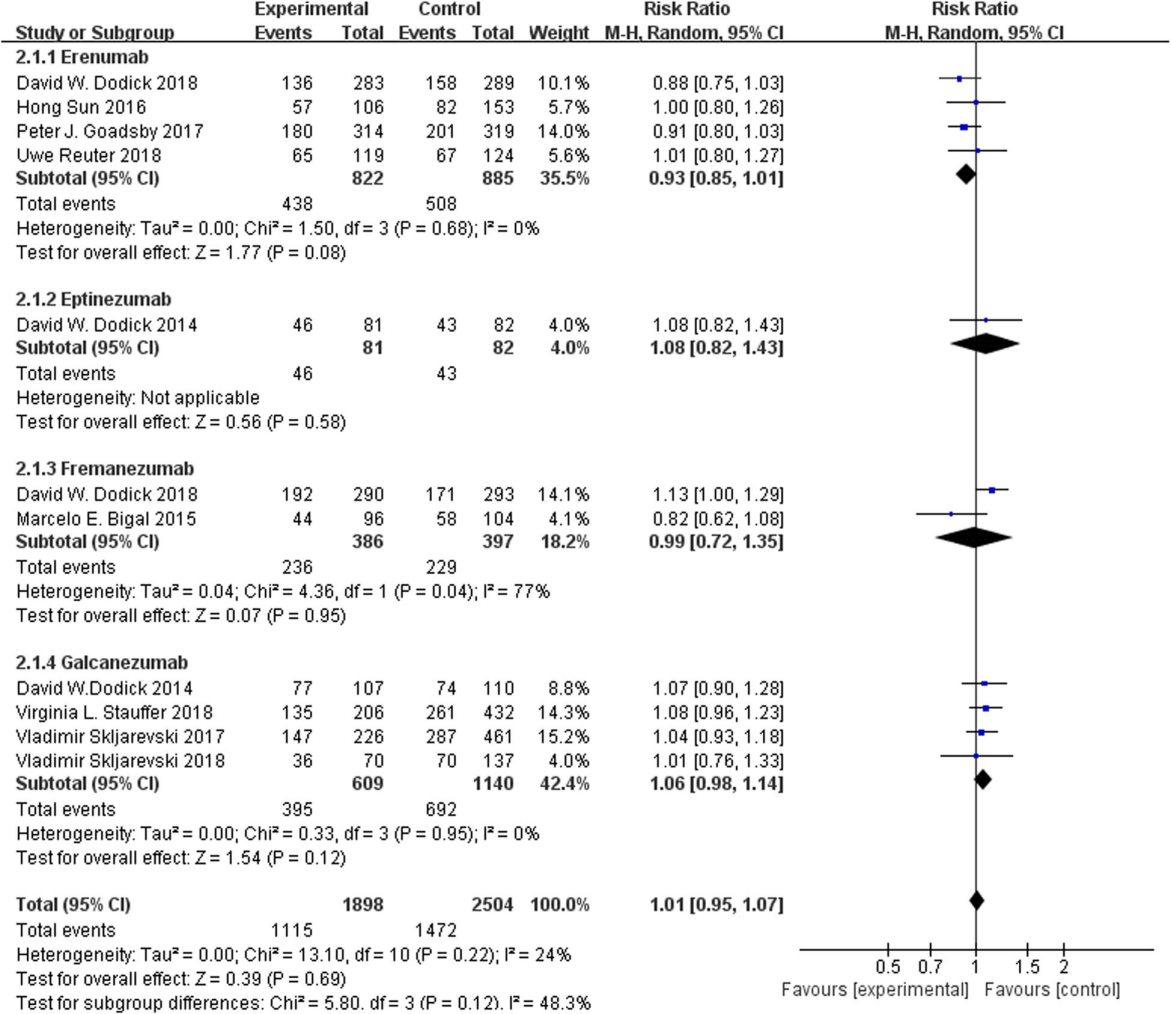
Forest plot of CGRP mAb vs. placebo for all types of adverse events. The estimated pooled RR was 1.01 (95% CI, 0.95 to 1.07) without statistical significance (*P* > 0.05). There was low heterogeneity among the studies (I^2^ = 24%). *CI* confidence interval, *RR* risk ratio

For the safety of CGRP mAb, the incidence of all types of AE was reported in the 11 studies. Regardless of pooled or subgroup analysis, the results demonstrated no significant difference between each CGRP mAb and placebo groups (Fig. 5).

Apart from AEs, we also assessed the treatment withdrawal rates due to AEs, incidence of SAEs and reported specific AEs. Of all the safety outcome measures, only the level of injection-site pain was significantly different between CGRP mAb and placebo groups (Table 3).

**Table 3:** Summary of adverse events among the included RCTs

	CGRP mAb(n/N)	Placebo(n/N)	I^2^	odds ratio [95% CI]	*p* value
Withdrawal due to AEs	38/1898	35/2504	0%	1.46[0.90,2.37]	0.12
Specific AEs					
any serious events	1115/1898	1472/2504	25%	1.02[0.90,1.15]	0.79
dizziness	29/835	31/1313	0%	1.47[0.87,2.49]	0.15
fatigue	36/1515	39/1825	0%	1.15[0.72,1.83]	0.55
influenza	26/1231	41/1758	5%	0.87[0.53,1.45]	0.6
injection site pain	167/1501	148/1837	35%	1.44[1.13,1.84]	**0.004**
migraine	12/1086	17/1379	11%	0.83[0.41,1.71]	0.62
nasopharyngitis	115/1817	163/2422	1%	0.96[0.75,1.24]	0.78
nausea	34/1553	61/1919	0%	0.68[0.45,1.05]	0.08
upper respiratory tract infection	117/1692	123/2072	0%	1.25[0.96,1.63]	0.1
urinary tract infection	22/1270	33/1519	0%	0.91[0.53,1.56]	0.73

### Trial sequential analysis

**Fig. 6:**
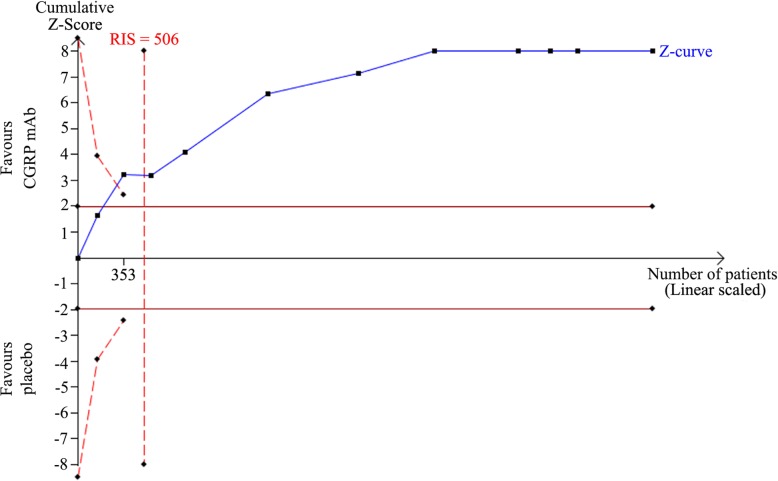
Random-effect model of trial sequential analysis for changes in monthly migraine days. The dashed red lines represent the trial sequential monitoring boundary (upper O’Brien Fleming with α = 5%, β = 20%, low risk of bias). Required information size (RIS) of 506 participants were calculated. Complete blue line represents cumulative Z-curve, which is well past the RIS needed. Cumulative Z-curve cross conventional boundary (complete red line) and the trial sequential monitoring boundary (dashed red line)

TSA was performed to evaluate random errors caused by limited data and repetitive testing of accumulating data. For the TSA, the required information size was calculated based on low risk of bias model. The type I error (α) was set at 0.05 and the power (1-β) at 0.80.The cumulative z-curve crossed both the traditional boundary and the trial sequential monitoring boundary, suggesting firm evidence for changes in monthly migraine days from baseline to endpoint (Fig. 6). Similarly, TSA supported sufficient evidence for changes in monthly acute migraine-specific medication days and ≥ 50% reduction in migraine days per month from baseline to endpoint (Additional file 1: Figure S1, S2).

### Publication bias

**Fig. 7:**
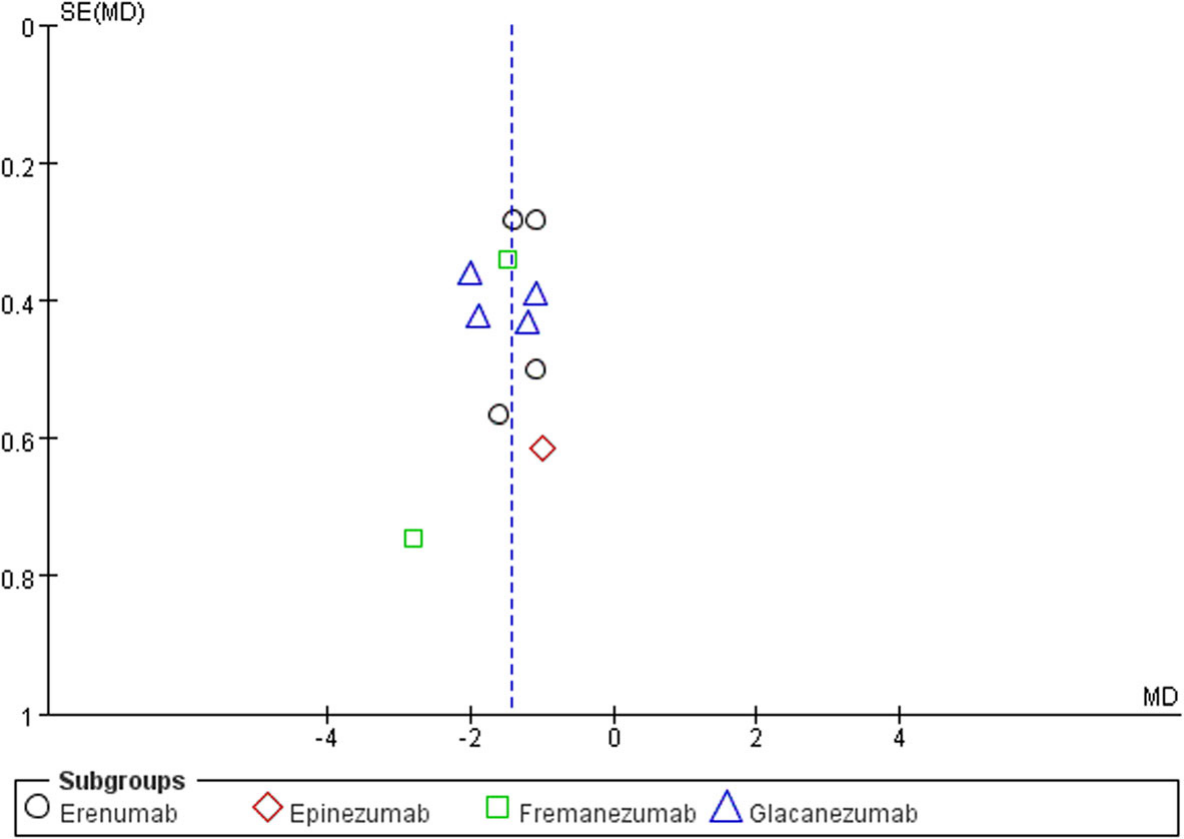
Funnel plot of effect size by standard error (surrogate for study size) across all studies. No obvious asymmetry was identified in the funnel plot, indicating that there was no publication bias. *SE* standard error, *MD* mean difference

A funnel plot of all studies (Fig. 7) explored the potential for publication bias in our sample. No obvious asymmetry was identified in the funnel plot, indicating that there was no publication bias.

## Discussion

In this meta-analysis of 11 high-quality studies involving a total of 4402 episodic migraineurs, we found that CGRP mAbs could reduce the numbers of monthly migraine days and acute migraine-specific medication days, as well as improve the 50% responder rate, as compared to placebo group. TSA was used to adjust random errors and calculate the sample size needed, and it was found that the evidence in our meta-analysis was reliable and conclusive. In addition, CGRP-binding mAbs were well tolerated among episodic migraineurs, as the incidence of AEs and treatment withdrawal rates were relatively similar between CGRP mAbs and placebo groups. Moreover, only injection-site pain was significantly different between CGRP mAbs and placebo groups. We speculated that it could be related to the subcutaneous delivery route of CGRP mAb administration. The outcomes of subgroup analysis revealed that erenumab, fremanezumab and galcanezumab exhibited similar efficacy and safety in patients with episodic migraine. Stephen D. Silberstein et al. [8] did a subgroup analysis of two phase 3 studies which we have included in our meta-analysis [17, 18] to evaluate the efficacy of galcanezumab for HFEM (8–14 monthly MHDs) and LFEM (4–7 monthly MHDs). And it was found that galcanezumab was as effective in patients with HFEM as in those with LFEM. Associated symptoms, quality of life, and disability were similarly improved in patients with HFEM or LFEM. While, the reported clinical information on eptinezumab are limited, resulting in only one study included for this mAb. A large multi-center RCT of eptinezumab, also known as PROMISE-1 (NCT02559895), has been completed recently. Still, more research is needed to confirm the treatment effects of eptinezumab on episodic migraine.

Compared to previous attempts [13, 31–33] aimed to summarize the evidence on CGRP mAb treatment in episodic migraine, this study provides a systematic, qualified, updated and more detailed assessment of the efficacy and safety of various CGRP mAbs. Indeed, this meta-analysis covered a greater number of studies and larger sample size, in order to obtain more precise estimates of the treatment effects. To the best of our knowledge, this is the first comprehensive study that includes 6 phase III trials to evaluate the efficacy and safety of CGRP-binding mAbs in patients with episodic migraine. The previous meta-analysis [13] published in 2018 is consisted of repeated trials and chronic migraine cases, leading to a doubtful conclusion. Another meta-analysis [33] recently published in 2019 contained a mixture of episodic and chronic migraineurs. Although the most recent meta-analysis has relatively similar included RCTs compared with our study, it mainly focused on the safety and tolerability rather than the efficacy of CGRP mAb in patients with episodic migraine [32].

In recent years, the new targets for migraine treatment are moving toward the trigeminal sensory neuropeptide CGRP or its receptor [34]. It’s reported that most of CGRP is released from trigeminal afferents both in meningeal tissues and at the first synapse in the spinal trigeminal nucleus [35]. And CGRP receptors are distributed in the central and peripheral nervous system, as well as in the cardiovascular system [36]. Since CGRP and mAbs cannot easily pass the blood-brain barrier, they may act in the trigeminal ganglion to influence the production of pronociceptive substances and receptors, which are transported along the central terminals into the spinal trigeminal nucleus. Therefore, mAbs against CGRP or CGRP receptor can have a central antinociceptive effect through a peripherally acting way [35]. However, the downstream molecular mechanisms following ligand-receptor blockade have not been clearly demonstrated. It’s indicated that inactivating CGRP by anti-CGRP antibodies or blocking CGRP access to trigeminal neurons by anti-CGRP receptor antibodies, can interrupt CGRP-induced cAMP accumulation and inhibits CGRP receptor internalization [37].CGRP-related drugs have numerous advantages over existing conventional therapies, as they are designed specifically to act on the trigeminal pain system, along with more specific mechanisms of action and fewer adverse effects. CGRP receptor antagonists, such as ubrogepant and so on, are effective in relieving acute migraine headache, but the underlying liver toxicity restricts their long-term usage [38, 39]. Since CGRP has important vasodilating effects and could protect organs from ischemia, the effect of CGRP blockade on cardiovascular system may be concerned. In the short- and long-term studies about animals and humans published, neither any hypertensive effect nor any negative effects regarding the development or aggravation of cardiac failure was observed [36]. Based on the findings of this meta-analysis, mAbs against CGRP (eptinezumab, fremanezumab and galcanezumab) and CGRP receptor (erenumab) could effectively prevent episodic migraine attacks without obvious adverse effects. However, the majority of results obtained from the included trials are achieved at 12 weeks or 24 weeks after treatment, and thus further trials are needed to determine the long-term safety of CGRP mAbs and the durability of their effects. A retrospective pooled analysis in chronic migraineurs was conducted to assess the effects of discontinuation of preventive erenumab and galcanezumab treatment. The results showed continuous efficacy of mAbs against CGRP/CGRP receptor in the prevention of chronic migraine up to 12 weeks after treatment discontinuation [40]. As for the differences in efficacy among the four mAbs, no direct comparison has ever been made, which requires a large RCT in the future.

Nevertheless, there are several limitations that need to be addressed. Firstly, different dosages of the same mAb were encompassed in the subgroup analysis, which might increase the between-study heterogeneity. For example, all the included studies for applied 70 mg of erenumab per month, with an exception of 140 mg per month in one RCT. Secondly, not all the outcome measures were from the same time point among the different trials. Most of the double-blind, placebo controlled trials lasted for 12 weeks, except for three studies with 24 weeks [17, 18, 24]. For the STRIVE trial, despite that the primary end point was the change in the mean number of monthly migraine days from baseline to months 4–6 [24],we extracted the supplemental data starting from the third month (i.e. 9–12 weeks) in order to enhance comparability. Moreover, since the original data were unretrievable, we could only extracted the outcome values at month 6 for two studies [17, 18]. Thirdly, different inclusion criteria could bias the results. For instance, the LIBERTY study included eligible participants who had previously been treated unsuccessfully (in terms of efficacy or tolerability, or both) with 2–4 conventional preventive therapies [14]. However, in the STRIVE trial, patients were excluded if they had no therapeutic response to more than two classes migraine preventive therapy [24].

## Conclusion

Our meta-analysis reveals that CGRP mAbs can serve as an effective and safe preventive treatment for episodic migraine.

## Supplementary information

**Additional file 1: Figure S1.** Random-effect model of trial sequential analysis for changes in monthly acute migraine-specific medication days. The dashed red lines represent the trial sequential monitoring boundary (upper O’Brien Fleming with α = 5%, β = 20%, low risk of bias). Required information size (RIS) of 1365 participants were calculated. Complete blue line represents cumulative Z-curve, which is well past the RIS needed. Cumulative Z-curve cross conventional boundary (complete red line) and the trial sequential monitoring boundary (dashed red line). **Figure S2.** Random-effect model of trial sequential analysis for changes in 50% reduction in migraine days per month. The dashed red lines represent the trial sequential monitoring boundary (upper O’Brien Fleming with α = 5%, β = 20%, low risk of bias and 34%control event rate (the control event rate in our meta-analysis)). Required information size (RIS) of 545 participants were calculated. Complete blue line represents cumulative Z-curve, which is well past the RIS needed. Cumulative Z-curve cross conventional boundary (complete red line) and the trial sequential monitoring boundary (dashed red line).

## Data Availability

The data is available on request to the corresponding author.

## Abbreviations

AEs: Adverse events
CENTRAL: The Cochrane Controlled Trials Register
CGRP mAb: Calcitonin-gene-related peptide binding monoclonal antibody
CIs: Confidence intervals
MD: Mean difference
PRISMA: Preferred Reporting Items for Systematic Reviews and Meta-Analyses
RCTs: Randomized control trials
RR: Risk ratio
TSA: Trial sequential analysis
WMD: Weighted mean difference
