# Supplementary figures and images for: Efficacy and safety of calcitonin-gene-related peptide binding monoclonal antibodies for the preventive treatment of episodic migraine – an updated systematic review and meta-analysis
Source: BMC Neurol. 2020 Feb 15;20:57. doi: 10.1186/s12883-020-01633-3 (PMC7023812; doi:10.1186/s12883-020-01633-3)

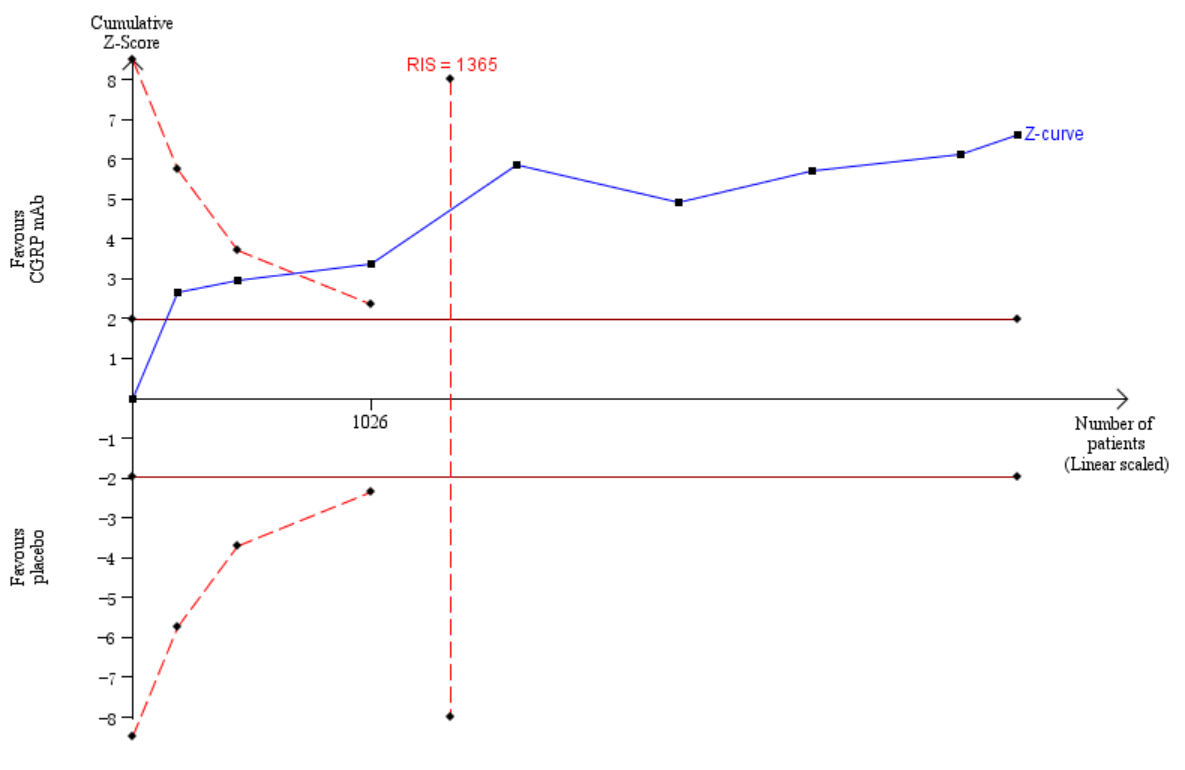

Supplement: Supplementary file 1 — Additional file 1: Figure S1. Random-effect model of trial sequential analysis for changes in monthly acute migraine-specific medication days. The dashed red lines represent the trial sequential monitoring boundary (upper O’Brien Fleming with α = 5%, β = 20%, low risk of bias). Required information size (RIS) of 1365 participants were calculated. Complete blue line represents cumulative Z-curve, which is well past the RIS needed. Cumulative Z-curve cross conventional boundary (complete red line) and the trial sequential monitoring boundary (dashed red line). Figure S2. Random-effect model of trial sequential analysis for changes in 50% reduction in migraine days per month. The dashed red lines represent the trial sequential monitoring boundary (upper O’Brien Fleming with α = 5%, β = 20%, low risk of bias and 34%control event rate (the control event rate in our meta-analysis)). Required information size (RIS) of 545 participants were calculated. Complete blue line represents cumulative Z-curve, which is well past the RIS needed. Cumulative Z-curve cross conventional boundary (complete red line) and the trial sequential monitoring boundary (dashed red line). [file 12883_2020_1633_MOESM1_ESM.zip › Supplementary Figure S1R2.tif]

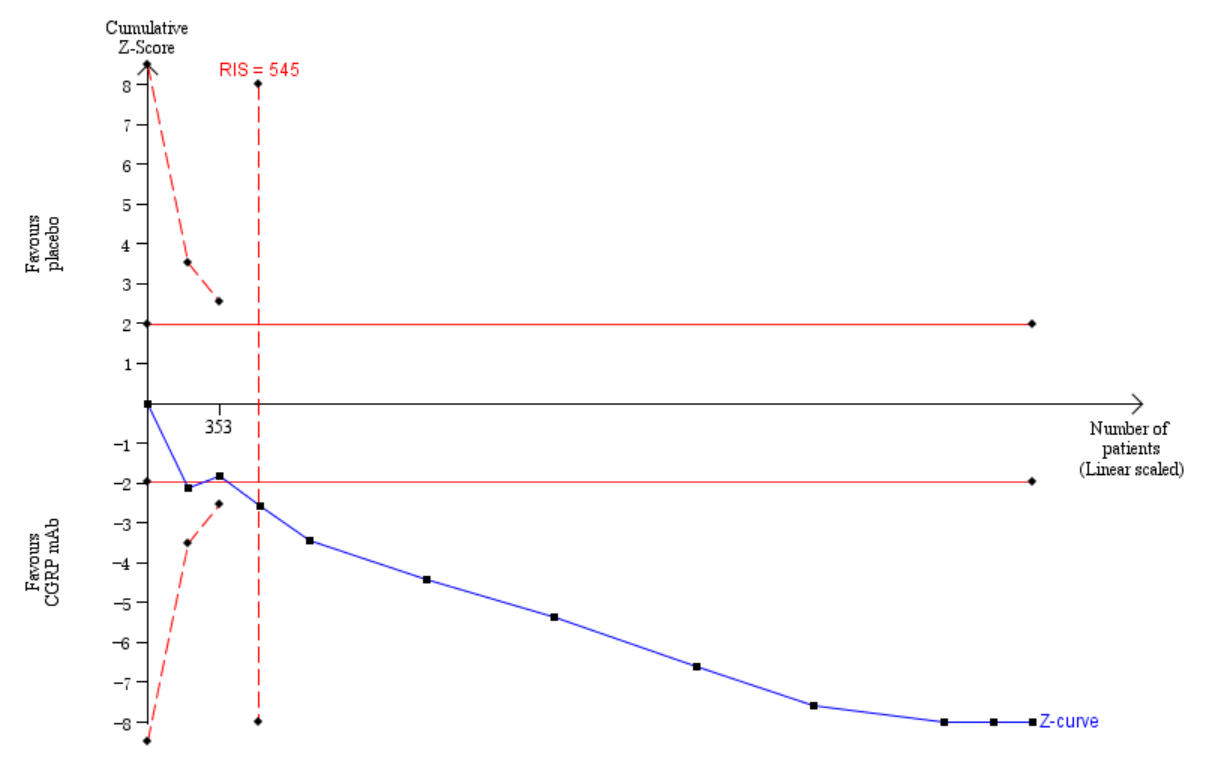

Supplement: Supplementary file 1 — Additional file 1: Figure S1. Random-effect model of trial sequential analysis for changes in monthly acute migraine-specific medication days. The dashed red lines represent the trial sequential monitoring boundary (upper O’Brien Fleming with α = 5%, β = 20%, low risk of bias). Required information size (RIS) of 1365 participants were calculated. Complete blue line represents cumulative Z-curve, which is well past the RIS needed. Cumulative Z-curve cross conventional boundary (complete red line) and the trial sequential monitoring boundary (dashed red line). Figure S2. Random-effect model of trial sequential analysis for changes in 50% reduction in migraine days per month. The dashed red lines represent the trial sequential monitoring boundary (upper O’Brien Fleming with α = 5%, β = 20%, low risk of bias and 34%control event rate (the control event rate in our meta-analysis)). Required information size (RIS) of 545 participants were calculated. Complete blue line represents cumulative Z-curve, which is well past the RIS needed. Cumulative Z-curve cross conventional boundary (complete red line) and the trial sequential monitoring boundary (dashed red line). [file 12883_2020_1633_MOESM1_ESM.zip › Supplementary Figure S2R2.tif]
